# A novel typing method for *Clostridium perfringens* using multiplex recombinase polymerase amplification and CRISPR/Cas12a

**DOI:** 10.3389/fmicb.2026.1770883

**Published:** 2026-02-18

**Authors:** Siying Li, Qinghong Zhou, Qingxun Zhang, Ziqin Lin, Qianyi Zhang, Sihong Wu, Luying Wang, Sheng Ye, Xingxing Xiao, Shuai Gao

**Affiliations:** 1 Wenzhou Key Laboratory of Sanitary Microbiology, Key Laboratory of Laboratory Medicine, Ministry of Education, School of Laboratory Medicine and Life Sciences, Wenzhou Medical University, Wenzhou, China; 2 Department of Laboratory Medicine, The Second Xiangya Hospital, Central South University, Changsha, China; 3 Beijing Milu Ecological Research Center, Beijing Academy of Science and Technology, Beijing, China; 4 Jingzhou Central Hospital, Jingzhou, China; 5 School of Sports Science, Wenzhou Medical University, Wenzhou, China; 6 Department of Clinical Laboratory, Affıliated Hospital of Jiaxing University, The First Hospital of Jiaxing, Jiaxing, China

**Keywords:** *Clostridium perfringens*, CRISPR/Cas12a, multiplex RPA, toxinotype, typing method

## Abstract

Distinct toxinotypes of *Clostridium perfringens* cause different diseases in animals and humans. Rapid and accurate typing methods remain essential for early diagnosis, effective intervention, and reduced mortality. In this study, we designed specific primer pairs and crRNA sequences targeting the α, β, ε, and ι toxin genes of *C. perfringens* and constructed a rapid, sensitive, accurate and instrument-free method for typing of *C. perfringens*. This typing method of *C. perfringens* based on the multiplex recombinase polymerase amplification (RPA)-assisted CRISPR/Cas12a system, termed Cp-MRC12a, can be completed in 1 h. The Cp-MRC12a assay shows high sensitivity with a detection limit of 10 copies/μL for the type A strain and 100 copies/μL for the type B-E strains and high specificity without cross-reactivity to non-target bacteria, and demonstrates a reliable performance in detecting clinical and spiked samples. Collectively, Cp-MRC12a provides a robust and practical approach for the typing of *C. perfringens* strains, offering substantial advances for early disease diagnosis and pathogen identification.

## 1 Introduction

*Clostridium perfringens*, a Gram-positive spore-forming anaerobic bacterium, is one of the most important pathogens responsible for histotoxic disease and intestinal infections in livestock and humans (Gohari et al., 2021; Lucey and Hutchins, 2004; Stevens et al., 2012). Its pathogenicity is largely driven by the production of twenty extracellular toxins and hydrolytic enzymes (Camargo et al., 2024; Gohari et al., 2021; Revitt-Mills et al., 2015). Among these, the four major lethal toxins—alpha (α), beta (β), epsilon (ε), and iota (ι) toxins—are key determinants of virulence (Hatheway, 1990), and form the basis for classifying *C. perfringens* into five toxinotypes (A-E) (Supplementary Table 1; Camargo et al., 2024; Gohari et al., 2021; Rood et al., 2018). All toxinotypes produce α toxin; additionally, type B strains also produce β and ε toxins, type C strains produce β toxin, type D strains produce ε toxin, and type E strains produce ι toxin (Gohari et al., 2021; McDonel, 1980; Petit et al., 1999). These toxinotypes are associated with distinct disease manifestations across host species (Supplementary Table 1; Gohari et al., 2021; Moustafa et al., 2022; Munday et al., 2020; Petit et al., 1999; Uzal et al., 2014). For example, both type A and C strains of *C. perfringens* can cause food poisoning, but type C infections are typically more severe and, without timely treatment, often fatal (Brynestad and Granum, 2002). Diseases caused by *C. perfringens* are characterized by diverse clinical presentation, rapid progression, and high mortality (Brynestad and Granum, 2002; Kiu and Hall, 2018; Wang et al., 2021; Zhong et al., 2023), posing a significant threat to public health and causing substantial economic losses in the livestock industry. Consequently, developing simple, rapid and accurate typing methods for the toxinotyping of *C. perfringens* is essential for timely diagnosis, effective intervention, and improved disease control.

At present, the typing methods for *C. perfringens* strains include antigen-antibody reaction-based methods (Hadimli et al., 2012; Yoo et al., 1997) and nucleic acid detection techniques (NADT) (Gurjar et al., 2008; Yoo et al., 1997). Traditionally, serum neutralization tests in mice or guinea pigs have been performed to classify *C. perfringens* strains (Yoo et al., 1997), but these methods are labor-intensive, costly, and no longer ethically acceptable (van Asten et al., 2009). ELISA-based assays provide an alternative, yet they often fail to simultaneously identify multiple toxins (Hadimli et al., 2012). With advances in NADT, several PCR-based methods, including multiplex PCR assays and real-time multiplex PCR assays, have been established to type *C. perfringens* strains (Gurjar et al., 2008; Yoo et al., 1997). However, these assays require specialized thermal cyclers and trained personnel, limiting their applicability in field diagnostics and resource-limited environments. Recent progress in isothermal nucleic acid amplification technology (iNAT) has led to the development of multiplex assays based on RPA (Crannell et al., 2016; Li et al., 2023; Ma et al., 2020) and LAMP (Iseki et al., 2007; Jang et al., 2021; Mahony et al., 2013), offering promising options for pathogen detection and typing in different settings. Nevertheless, these methods share drawbacks with their uniplex counterparts: LAMP-based multiplex methods are hindered by complex primer design, while RPA-based multiplex methods often require complicated result interpretation (Craw and Balachandran, 2012; Nagura-Ikeda et al., 2020). Therefore, there remains a critical need for a rapid, accurate, and instrument-free typing method to support timely control and prevention of *C. perfringens* infections.

The discovery of the *trans*-cleavage activity of CRISPR/Cas (Clustered Regularly Interspaced Short Palindromic Repeats and CRISPR-associated protein) systems has revolutionized molecular diagnostics, offering powerful capabilities for pathogen detection (Chen et al., 2018; Kaminski et al., 2021; Wang and Doudna, 2023). Leveraging preamplification-assisted CRISPR/Cas platforms, several detection systems—such as DETECTR (Chen et al., 2018), SHERLOCK (Gootenberg et al., 2018), and HOLMES (Li et al., 2018)—have been developed, providing rapid, sensitive, specific, and instrument-free detection suitable for disease diagnosis and pathogen identification (Kaminski et al., 2021). Building on these advances, many teams including ours have established pathogen detection methods by using RPA assay and CRISPR/Cas12a system (Bhattacharyya et al., 2018; Wang et al., 2026; Xiao et al., 2023; Yang et al., 2024). Moreover, we recently developed a multiplex RPA-assisted CRISPR/Cas12a platform, termed MARPLES (Multiplex Assay of RPA and Collateral Effect of Cas12a-based System), and demonstrated its applicability in diagnosing the hand, foot, and mouth disease and identifying the pathogen of influenza A (Lin et al., 2023). MARPLES enables rapid, specific, sensitive, and simultaneous detection of multiple target genes, highlighting its strong potential for pathogen identification. Thus, to better identify the different toxinotype of *C. perfringens* strains, we employed the MARPLES platform to develop a dedicated typing assay.

**FIGURE 1:**
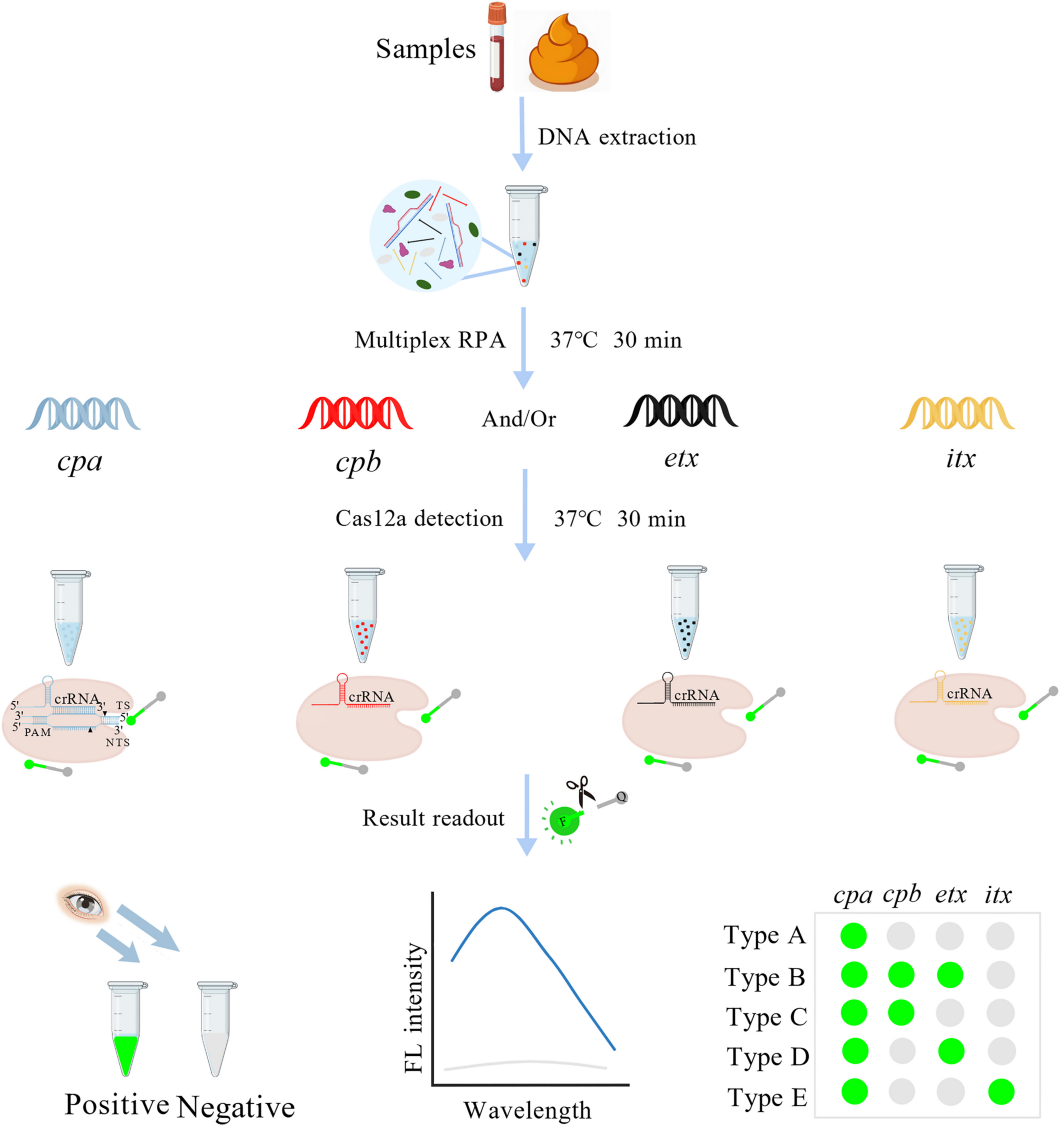
Schematic diagram of the established Cp-MRC12a method in the detection and typing of *C. perfringens*. F, fluorophore; Q, quencher; FL, fluorescence.

In this study, we established a rapid, accurate, and instrument-free typing method for discriminating the five toxinotypes (A-E) of *C. perfringens* based on a multiplex RPA-assisted CRISPR/Cas12a assay (Figure 1), termed Cp-MRC12a. The Cp-MRC12a assay exhibits high sensitivity for detecting genomic DNA from all five toxinotypes and demonstrates excellent specificity with no cross-reactivity to non-target bacteria. It also performs robustly in detecting clinical samples and spiked animal samples. The entire workflow can be completed within one hour. Collectively, the Cp-MRC12a assay provides a novel and practical platform for early diagnosis and toxinotype identification of *C. perfringens* in both humans and animals, with particular value for use in resource-limited or field settings.

## 2 Materials and methods

### 2.1 Bacterial strains, plasmids, and genomic DNA extraction

Fourteen bacterial strains (12 reference strains and two isolation strains) were used in this study. Twelve reference strains included *C. perfringens* Type A (CVCC 2015), *C. perfringens* Type B (CVCC 54), *C. perfringens* Type C (CVCC 1153), *C. perfringens* Type D (CVCC 60201), *C. perfringens* Type E (CVCC 90), *Pseudomonas aeruginosa* (ATCC 27853), *Escherichia coli* (ATCC 25922), *Aeromonas hydrophila* (ATCC 7966), *Staphylococcus aureus* (ATCC 25923), *Vibrio vulnificus* (ATCC 27562), *Bacillus cereus* (ATCC 14579), and *V. harveyi* (ATCC 14126). Two isolation strains included *V. parahaemolyticus* and *Salmonella typhimurium*. Five *C. perfringens* reference strains were purchased from the China Veterinary Culture Collection Center and served as positive controls for developing the Cp-MRC12a assay. To investigate the specificity of our established method in typing *C. perfringens* strains, nine non-*C. perfringens* bacteria were selected as negative controls.

Four recombinant plasmids (pMD-19T-*cpa*, pMD-19T-*cpb*, pMD-19T-*etx*, and pMD-19T-*itx*) containing the toxin gene of *C. perfringens* were constructed and subsequently extracted using the E.Z.N.A.^®^ Plasmid Mini Kit I (OMEGA Bio-Tek, United States). Genomic DNA extraction was performed from bacterial cultures using the MiniBEST Bacteria Genomic DNA Extraction Kit Ver.3.0 (TaKaRa Bio Inc., Japan). These DNA samples were used in the following assays.

### 2.2 Design of RPA primer pairs and crRNA

The selection of RPA primer pairs and crRNA sequences is very important for the specificity and sensitivity of the Cp-MRC12a assay. To establish a Cp-MRC12a assay with high sensitivity and specificity, several candidate RPA primer pairs and crRNA sequences targeting the *C. perfringens cpa*, *cpb*, *etx*, and *itx* genes were designed according to the principle mentioned in our previous publication (Xiao et al., 2021). In brief, several sequences for each toxin gene were downloaded from GenBank and subsequently aligned to identify the conserved regions for designing RPA primers and crRNAs. Then, the RPA primer pairs were designed using the NCBI Primer-BLAST tool, and the crRNA sequences were designed using the online software EasyDesign^1^ (Huang et al., 2024).

The designed RPA primer pairs and crRNA sequences were then screened to obtain the optimal combination of the RPA primer pair and crRNA for each toxin gene according to the guidelines from our previous publications (Xiao et al., 2023; Yang et al., 2024). Briefly, the uniplex RPA assays were performed using each primer pair and its corresponding recombinant plasmid. RPA products were purified using the PCR Clean-up Kit (UElandy, China) and then analyzed by 2% agarose gel electrophoresis to screen primer pairs. Next, different combinations of the screened RPA primer pair and the designed crRNA were valuated using RPA-CRISPR/Cas12a assays to verify the feasibility of crRNA sequences and investigate the efficiency of each combination. Finally, we obtained four optimal combinations of RPA primer pair and crRNA targeting the *C. perfringens cpa*, *cpb*, *etx*, and *itx* genes, respectively (shown in Table 1) and then used them in the following assays.

**TABLE 1:** Sequences of primer pairs for multiplex RPA and crRNA for Cp-MRC12a in this study.

Toxin (gene)	Primer sequence (5′–3′)	Product length (bp)	crRNA sequence (5′–3′)
α (*cpa*)	AF: TAAAGTCTACGCTTGGGATGGAAAGATTGA AR: TATATCTCCAAAATAGTGCATAGCCTCTCC	406	CCA UUC AUA UCU AGC UAA U
β (*cpb*)	BF: TTCTACTATACAGACAGATCATTCAACCTC BR: TTATAGATTCTTCAGTACCATTAGGAGCAG	260	UGU ACG GAA GAU AUA CUA A
ε (*etx*)	EF: GAAATGTAAAGTTAGTAGGACAAGTAAGTGG ER: CTTAACTATTAACTCATCTCCCATAACTGC	197	AAU GAA GAU GGU ACU AUU A
ι (*itx*)	IF: TGAACTTGCTGATGTAAATGACTATATGCG IR: GGAGATGTGAGAGTTAATCCAAATTCTTGTG	213	AUA UUA AAU AGU UAU UAA U

### 2.3 Uniplex and multiplex RPA assays

The uniplex RPA assay was performed using the RPA basic kit (Jiangsu Qitian Gene Biotechnology, China) for primer screening. The following components were added sequentially to the lyophilized powder tubes: 25 μL of buffer V, 15 μL of purified water, 2 μL of forward primer (10 μM), 2 μL of reverse primer (10 μM), 5 μL of Magnesium Acetate I, and 1 μL of plasmid template. The obtained solution was thoroughly mixed and incubated at 37°C for 30 min.

The multiplex RPA assay was constructed using the four primer pairs obtained through the uniplex RPA screening. The reaction system of the multiplex RPA assay was similar to that of the uniplex RPA assay, with adjustments only in the volume of purified water, the number and usage of primers, and the template usage. Furthermore, the usage of primers should be optimized to amplify all target sequences robustly. The quadruplex RPA assay was conducted with a reaction mixture containing the following components: 25 μL of buffer V, 7.8 μL of purified water, 1.05 μL of α-F (10 μM), 1.05 μL of α-R (10 μM), 1.1 μL of β-F (10 μM), 1.1 μL of β-R (10 μM), 1.1 μL of ε-F (10 μM), 1.1 μL of ε-R (10 μM), 1.1 μL of ι-F (10 μM), 1.1 μL of ι-R (10 μM), 5 μL of Magnesium Acetate I, and 3.5 μL of DNA template. The mixture was thoroughly mixed and incubated at 37°C for 30 min. The RPA products were finally analyzed by 2% agarose gel electrophoresis or directly used in the Cas12a-mediated trans-cleavage assay.

### 2.4 Multiplex RPA-CRISPR/Cas12a assay

The multiplex RPA-CRISPR/Cas12a assay, comprising a multiplex RPA assay step and a Cas12a-mediated trans-cleavage assay step, was carried out according to the procedure of Scheme 2 mentioned in our previous publication (Lin et al., 2023). Briefly, four Cas12a-mediated trans-cleavage assays were performed simultaneously to detect the target genes of α, β, ε, and ι toxins from the products of multiplex RPA assay. For *cpa* gene detection, 10 μL of 200 nM Cas12a (New England Biolabs, United States) and 10 μL of 200 nM ACR were pre-incubated at 37°C for 20 min to form the Cas12a-crRNA complex. Subsequently, 10 μL of 500 nM ssDNA-FQ reporter (5′-/6-FAM/TTATT/BHQ1/-3′) and 2.5 μL of multiplex RPA products were added to the complex. The 32.5 μL reaction mixture was then incubated at 37°C for 30 min. Upon completion of the reaction, the fluorescence signal was measured by a UV torch or by a multifunctional microplate reader (λ_ex_: 490 nm, λ_em_: 522 nm). As for *cpb*, *etx* and *itx* gene detection, only the crRNA used was different from *cpa* gene detection, which was BCR, ECR, and ICR, respectively.

### 2.5 Multiplex PCR assay

The multiplex PCR assay, serving as the standard method to compare with our newly developed assay, was performed to detect the *cpa*, *cpb*, *etx*, and *itx* genes using previously designed primers (Supplementary Table 2; Ali et al., 2024; Hussain et al., 2022; Mohiuddin et al., 2020). The multiplex PCR reaction mixture contained 10 μL of Premix Extaq™ (TaKaRa Bio Inc., Japan), 1 μL (10 μM) of forward and reverse primers of *cpa*, *cpb*, *etx*, and *itx* genes, 0.5 μL of ddH_2_O, and 1.5 μL of DNA template. The mixture was thoroughly mixed and subjected to the following reaction conditions: 98°C for 5 min, 30 cycles of 98°C for 10 s, 58°C for 30 s, and 72°C for 30 s, 72°C for 10 min, and 4°C for 5 min. The results of the PCR assay were analyzed by gel electrophoresis.

### 2.6 Clinical and spiked sample analysis

To evaluate the practicability of the Cp-MRC12a assay, clinical stool samples were collected from five patients diagnosed with *C. perfringens* infection. Considering the epidemic characteristics of *C. perfringens* in animals, we selected animal fecal samples from four species (cattle, sheep, pig, and chicken) for spiked sample analysis. To prepare spiked samples, 10 μL of cattle fecal genomic DNA was added to two tubes containing 10 μL of *C. perfringens* type A or type D genomic DNA (1 × 10^4^ copies/μL), respectively, 10 μL of sheep fecal genomic DNA was added to the other two tubes containing 10 μL of *C. perfringens* type A or type D genomic DNA (1 × 10^4^ copies/μL), respectively, and 10 μL of pig fecal genomic DNA and 10 μL of chicken fecal genomic DNA were mixed with 10 μL of *C. perfringens* type A genomic DNA (1 × 10^4^ copies/μL), respectively. Subsequently, the five clinical samples, four non-spiked samples, and six spiked samples were subjected to the Cp-MRC12a assay and multiplex PCR assay. The results of the Cp-MRC12a assay and the multiplex PCR assay were compared to validate the reliability of the Cp-MRC12a assay.

### 2.7 Statistical analysis

Statistical analysis was performed using SPSS 13.0 software (SPSS Inc., Chicago, IL, United States). The data were analyzed using Student’s *t*-test, and a *p* < 0.05 (indicated by *) was considered statistically significant.

## 3 Results

### 3.1 Construction of the multiplex RPA assay

**FIGURE 2:**
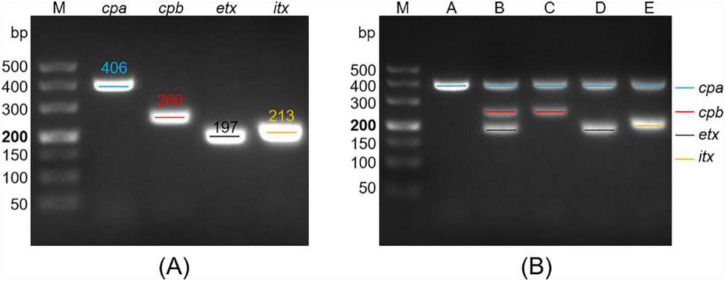
Construction of the multiplex RPA assay for amplifying the *cpa*, *cpb*, *etx*, and *itx* genes of *C. perfringens*. **(A)** Electrophoresis analysis of uniplex RPA products. Four uniplex RPA assays were performed using the screened primer pairs of *cpa*, *cpb*, *etx*, and *itx* genes and their respective recombinant plasmids as the template. **(B)** Electrophoresis analysis of multiplex RPA products. Five multiplex RPA assays were performed using the genomic DNA of *C. perfringens* type A, B, C, D, and E as the template, respectively. RPA products were analyzed using 2% agarose gel electrophoresis. M, 500 DNA marker.

To enable toxinotype identification of *C. perfringens* using a multiplex RPA-CRISPR/Cas12a workflow, we first established a multiplex RPA assay capable of simultaneously amplifying the *cpa*, *cpb*, *etx*, and *itx* genes. Multiple candidate primer pairs were designed for each toxin gene and evaluated using uniplex RPA reactions to screen the optimal primer set for each target (data not shown). Four primer pairs with superior amplification performance were ultimately selected (Table 1). As shown in Figure 2A, each uniplex RPA reaction generated a distinct band of the expected size, confirming high amplification efficiency. These four primer pairs were then combined to construct the multiplex RPA assay.

To construct a robust multiplex RPA assay, we next optimized the concentration of each primer pair. Multiplex RPA assays were conducted using different primer concentrations, and products were assessed by 2% agarose gel electrophoresis. Iterative optimization was terminated until the multiplex RPA products for each *C. perfringens* toxinotype showed clearly visible gel band(s). The final reaction conditions consisted of 1.05 μL (10 μM each) of α-F and α-R, respectively, 1.1 μL (10 μM each) of β-F, β-R, ε-F, ε-R, ι-F, and ι-R, respectively, and 3.5 μL of *C. perfringens* genomic DNA. Under these conditions, each multiplex RPA reaction produced the expected target band(s) (Figure 2B), demonstrating the feasibility of our constructed multiplex RPA assay for typing *C. perfringens*.

### 3.2 Establishment of the Cp-MRC12a assay

**FIGURE 3:**
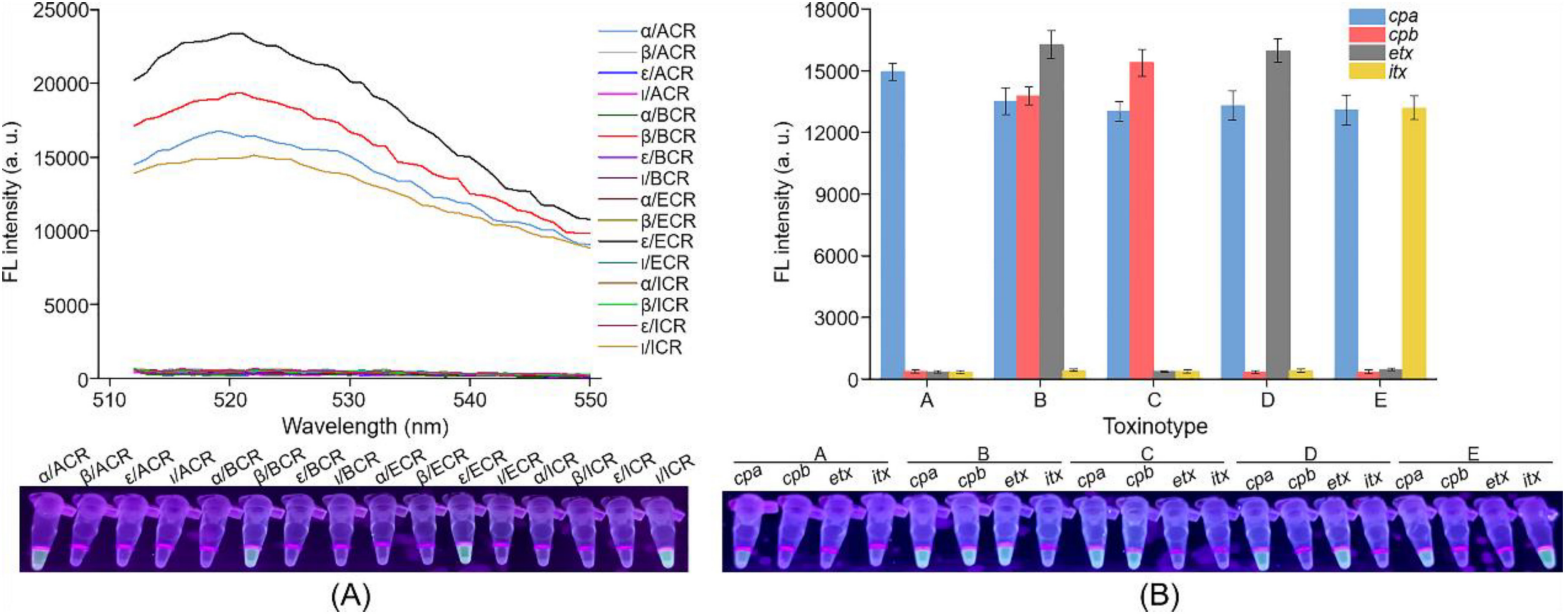
Establishment of the Cp-MRC12a assay for typing *C. perfringens*. **(A)** Feasibility analysis of ACR, BCR, ECR, and ICR in detecting their respective target genes. Four uniplex RPA assays were conducted to amplify the genes of α, β, ε, and ι, and then each RPA product was detected by CRISPR/Cas12a system using ACR, BCR, ECR, and ICR, respectively. **(B)** Feasibility analysis of the Cp-MRC12a assay in typing *C. perfringens*. The multiplex RPA-CRISPR/Cas12a assay was conducted using the genomic DNA of *C. perfringens* types A to E as the template to investigate whether the Cp-MRC12a assay can type *C. perfringens* accurately. Fluorescence signals were read by a multifunctional microplate reader (upper) or a UV flashlight (below).

To develop a simple, rapid and accurate method for typing *C. perfringens*, the Cp-MRC12a assay was established (Figure 1). Upon construction of the multiplex RPA assay for amplifying *C. perfringens* toxin genes, we further constructed the CRISPR/Cas12a system to detect the target sequences of *cpa*, *cpb*, *etx*, and *itx* genes. Several candidate crRNA sequences were designed for each gene and screened to identify those that most effectively activated Cas12a (data not shown). Four optimal crRNAs—ACR, BCR, ECR, and ICR—corresponding to *cpa*, *cpb*, *etx*, and *itx*, respectively, were selected (Table 1). Then, four uniplex RPA reactions were performed using recombinant plasmids containing the individual toxin genes as templates, and the resulting amplicons served as targets for four Cas12a-mediated *trans*-cleavage reactions using ACR, BCR, ECR, and ICR, respectively. As expected, each crRNA specifically recognized its corresponding target without cross-reactivity to the other three non-target genes (Figure 3A).

Subsequently, we evaluated the feasibility of the Cp-MRC12a assay in typing *C. perfringens*. Multiplex RPA reactions were performed using genomic DNA from *C. perfringens* types A to E as templates, followed by four Cas12a-mediated *trans*-cleavage reactions to determine which toxin genes were present in each amplified product. As shown in Figure 3B, fluorescence signals were generated exclusively by the toxin gene(s) corresponding to each specific toxinotype, demonstrating that the Cp-MRC12a assay enables accurate and effective typing of *C. perfringens*.

### 3.3 Sensitivity evaluation of the Cp-MRC12a assay

**FIGURE 4:**
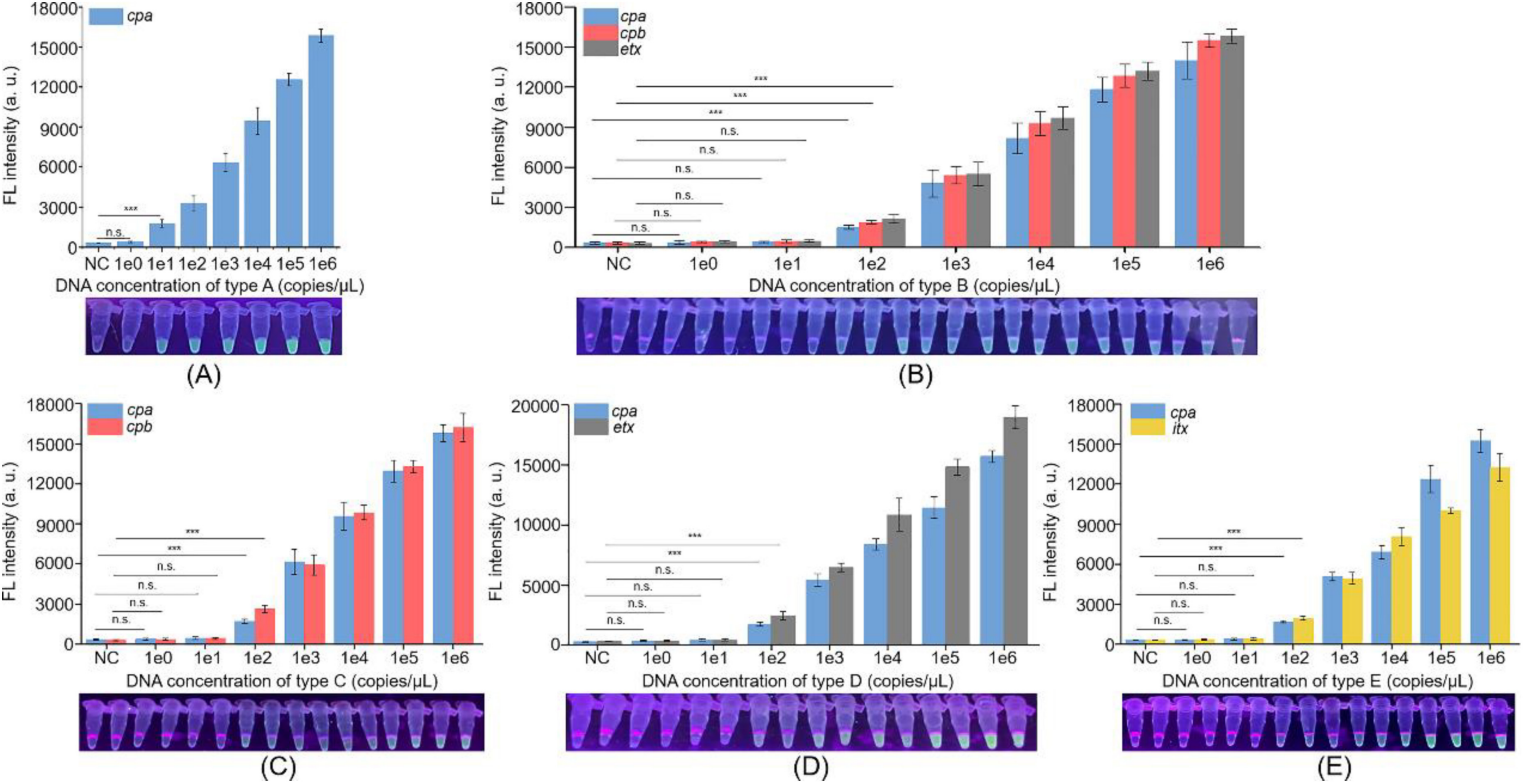
Sensitivity evaluation of the Cp-MRC12a assay in typing *C. perfringens*. Cp-MRC12a assays were conducted using the different concentrations of genomic DNA of *C. perfringens* type A **(A)**, type B **(B)**, type C **(C)**, type D **(D)**, and type E **(E)** as templates, and H_2_O was used as the negative control (NC). *n* = 3 technical replicates, two-tailed Student’s *t*-test; ***p < 0.001, the difference between sample group and respective H_2_O group (only shown the 1e0 vs. H_2_O and 1e1 vs. H_2_O for *C. perfringens* type A, and shown the 1e0 vs. H_2_O, 1e1 vs. H_2_O, and 1e2 vs. H_2_O for types) **(B–E)**; bars represent mean ± SEM. Fluorescence signals were read by a multifunctional microplate reader (upper) or a UV flashlight (below).

To evaluate the sensitivity of the Cp-MRC12a assay in typing *C. perfringens*, genomic DNA of each *C. perfringens* type was serially diluted to concentrations ranging from 10^0^ to 10^6^ copies/μL and used to evaluate the limit of detection (LOD) of this assay. For each reaction, 3.5 μL of genomic DNA or an equivalent volume of nuclease-free water was added as the sample input. For toxinotype A, fluorescence signals were detectable at DNA concentrations as low as 10^0^ copies/μL, whereas no signal was observed at 10^2^ copies/μL or in the negative control (Figure 4A). For toxinotypes B-E, fluorescence signals were consistently detected at concentrations down to 10^2^ copies/μL (Figures 4B–E). These results indicate that the Cp-MRC12a assay achieves an LOD of 10 copies/μL for toxinotype A and 100 copies/μL for toxinotypes B-E.

Furthermore, we also investigated the sensitivity of the multiplex PCR assay using the same samples as the Cp-MRC12a assay. The results showed that the LOD of the multiplex PCR method reached 10^3^ copies/μL for the *C. perfringens* A to E types (Supplementary Figure 1). Compared with this performance, the markedly lower LOD obtained using Cp-MRC12a demonstrates that the Cp-MRC12a assay exhibits substantially higher sensitivity for typing of *C. perfringens*.

### 3.4 Specificity investigation of the Cp-MRC12a assay

**FIGURE 5:**
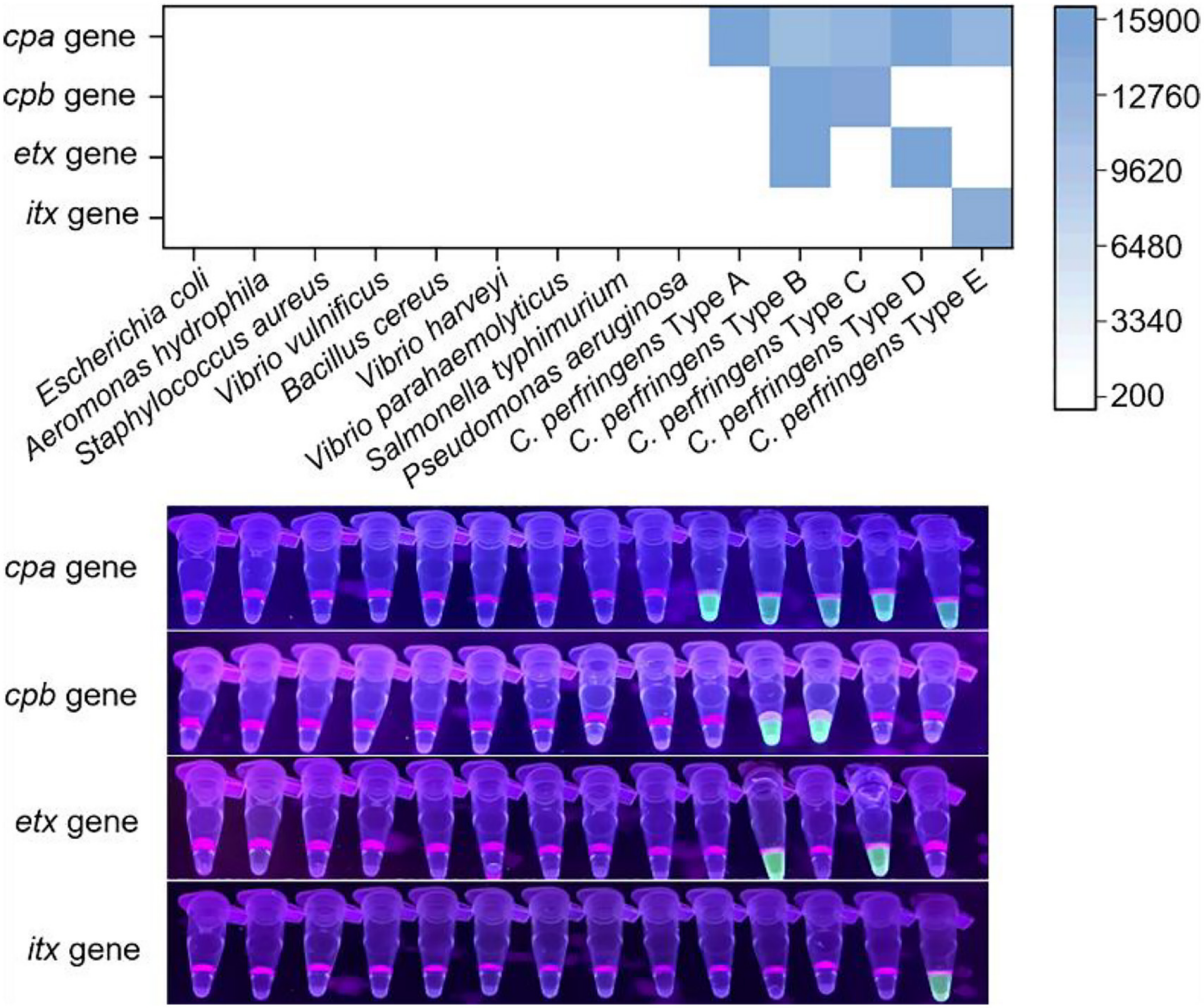
Specificity investigation of the Cp-MRC12a assay in typing *C. perfringens.* Nine non-*C. perfringens* bacteria and five types (A–E) of *C. perfringens* were detected by the Cp-MRC12a assay. The results were read by a multifunctional microplate reader or a UV flashlight. The heatmap represents the mean fluorescence values.

To investigate the specificity of the Cp-MRC12a assay in typing *C. perfringens*, genomic DNA from five *C. perfringens* toxinotypes (A-E) and nine non-*C. perfringens* bacterial pathogens was tested, with the latter serving as negative controls. As shown in Figure 5, fluorescence signals were generated exclusively in reactions containing *C. perfringens* DNA, whereas no signal was detected from any of the non-*C. perfringens* strains. Furthermore, each toxinotype produced fluorescence only in response to its corresponding target toxin gene(s), without cross-activation by non-target crRNAs. These results indicated that the Cp-MRC12a assay shows an excellent specificity, displaying no cross-reactivity with related bacterial species and accurately identifying all five *C. perfringens* toxinotypes.

### 3.5 Practicability assessment of the Cp-MRC12a assay in clinical and spiked samples

**FIGURE 6:**
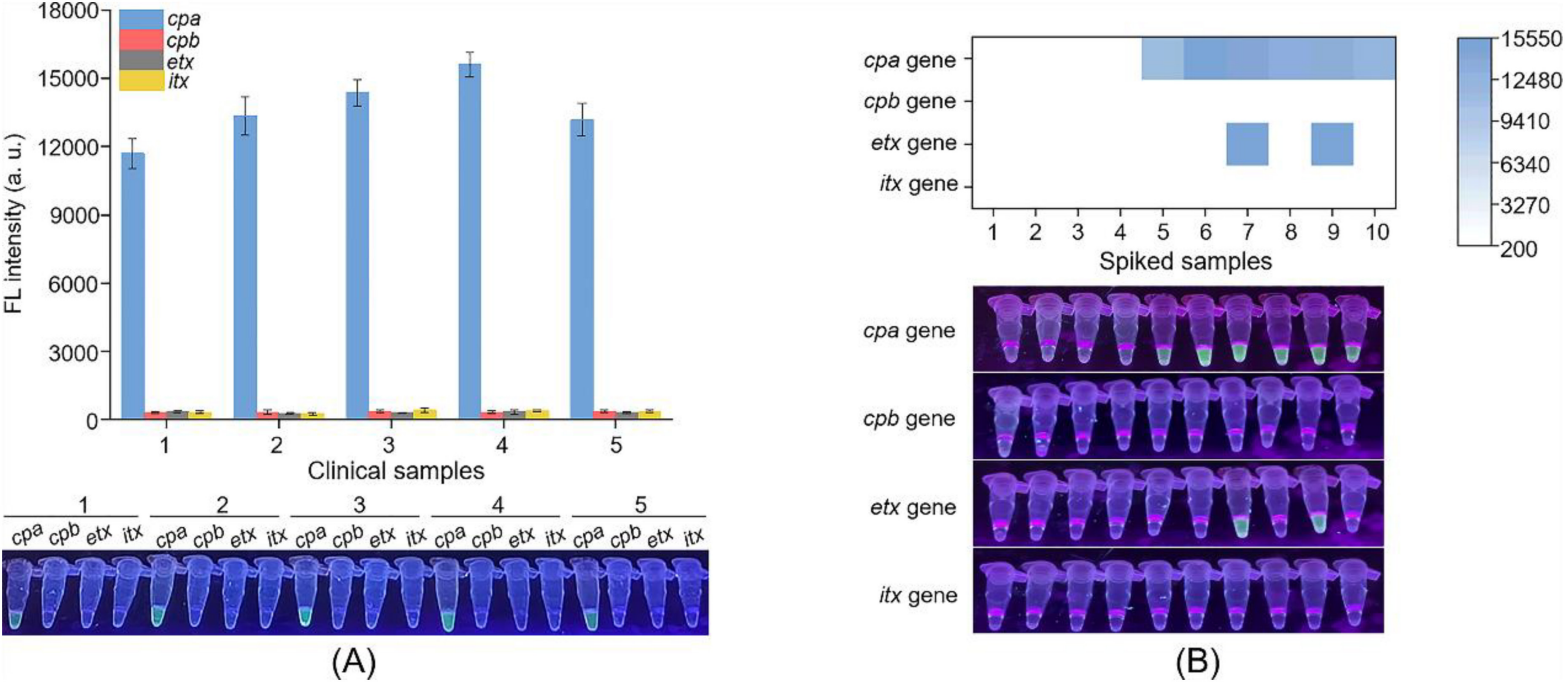
Practicability assessment of the Cp-MRC12a assay in typing *C. perfringens.* Five clinical samples **(A)** and six spiked samples **(B)** were analyzed by Cp-MRC12a. **(A)** 1–5 were five stool samples collected from five patients diagnosed with *C. perfringens* infection. *n* = 3 technical replicates; bars represent mean ± SEM. **(B)** 1–4 were four fecal samples collected from cattle, sheep, pig and chicken, respectively, 5 and 10 were pig and chicken samples spiked with *C. perfringens* type A genomic DNA, 6 and 7 were cattle samples spiked with genomic DNA of *C. perfringens* types A and D, respectively, and 8 and 9 were sheep samples spiked with genomic DNA of *C. perfringens* types A and D, respectively. The heatmap represents the mean fluorescence values.

To evaluate the practicality of the Cp-MRC12a assay, we evaluated its performance using clinical and spiked samples. Five clinical samples were collected from patients diagnosed with *C. perfringens* infection. As shown in Figure 6A, the Cp-MRC12a assay successfully detected *C. perfringens* in all samples and identified each as toxinotype A. In addition, to further increase sample diversity, six spiked samples—prepared by mixing animal genomic DNA with *C. perfringens* genomic DNA—and four non-spiked samples containing only animal genomic DNA were analyzed. As shown in Figure 6B, all spiked samples produced clear fluorescence signals corresponding to the expected toxinotypes, whereas no signals were observed in the non-spiked controls. These findings demonstrate that the Cp-MRC12a assay performs reliably in complex sample matrices and is suitable for practical typing of *C. perfringens*.

In parallel, the same set of samples was also analyzed using the multiplex PCR assay as a reference method. As shown in Supplementary Figure 2, five clinical samples were also identified as type A strains (Supplementary Figure 2A) and six spiked samples were correctly typed according to their respective spiked toxinotypes (Supplementary Figure 2B). These results indicate strong concordance between the Cp-MRC12a assay and the multiplex PCR assay. Collectively, these findings confirm that the Cp-MRC12a assay is suitable for detecting and typing *C. perfringens* in both human clinical samples and livestock-derived specimens.

In summary, the Cp-MRC12a assay for *C. perfringens* detection and typing provides significant advantages over existing methods, enabling rapid, sensitive, specific, and instrument-free typing of *C. perfringens* in clinical samples and spiked animal samples.

## 4 Discussion

*C. perfringens*, a highly versatile pathogen responsible for various diseases, is classified into five toxinotypes (A-E) based on its production of α, β, ε, and ι toxins (Camargo et al., 2024; Gohari et al., 2021; Rood et al., 2018), and various toxinotypes are associated with distinct diseases, many of which are characterized by rapid onset, high virulence, and substantial mortality (Brynestad and Granum, 2002; Kiu and Hall, 2018; Wang et al., 2021; Zhong et al., 2023). Consequently, the development of simple, rapid, and accurate typing methods is essential for protecting human and animal health and for mitigating disease transmission. Current approaches for *C. perfringens* typing include immunological assays (Hadimli et al., 2012; Yoo et al., 1997) and NADT such as multiplex PCR (Yoo et al., 1997) and real-time multiplex PCR (Gurjar et al., 2008). However, these techniques often suffer from limitations including lengthy workflows, operational complexity, reliance on specialized instrumentation, and suboptimal sensitivity. With the discovery of the collateral *trans*-cleavage activity of Cas12a, CRISPR/Cas12a system has become a popular nucleic acid detection technology for disease diagnosis and pathogen identification (Chen et al., 2018; Ge et al., 2025; Kaminski et al., 2021). Based on this system, we have previously developed a MARPLES platform which supports rapid, sensitive, specific, and multiplex nucleic acid detection and is broadly applicable across diverse pathogens (Lin et al., 2023). Therefore, to provide a more appropriate typing method for *C. perfringens*, we developed a novel detection and typing method of *C. perfringens*, Cp-MRC12a, based on the MARPLES platform (Figure 1).

In this study, we designed and screened the specific primer pairs and crRNA sequences (Table 1) targeting the *cpa*, *cpb*, *etx*, and *itx* genes and established the Cp-MRC12a assay (Figures 2, 3). This assay demonstrated time-saving and user-friendly operation, high sensitivity (Figure 4), and high specificity (Figure 5), allowing accurate typing of all five toxinotypes (A-E). The Cp-MRC12a assay achieved a LOD of 10 copies/μL for type A and a LOD of 100 copies/μL for the other four types (Figure 4). Although uniplex RPA-CRISPR/Cas12a assays typically outperform multiplex formats in analytical sensitivity (Ge et al., 2025; Xiao et al., 2023), multiplex systems offer the distinct advantage of detecting multiple targets simultaneously. In addition, the Cp-MRC12a assay exhibited higher sensitivity than multiplex PCR (Figure 4 and Supplementary Figure 1; van Asten et al., 2009; Yoo et al., 1997). The reduced sensitivity observed in multiplex RPA reactions is generally attributed to competition among primer sets for shared substrates and templates, which compromises amplification efficiency. Accordingly, in the multiplex RPA system, primer concentration becomes a critical determinant of assay performance, and the use of an optimal combination of primer pair and crRNA sequence for each gene is essential to ensure strong and reliable Cas12a activation. Here, we systematically optimized primer concentrations and identified the most effective primer-crRNA combination for each target gene. As a result, the Cp-MRC12a assay achieved high specificity, showing no cross-reactivity with nine non-*C. perfringens* strains and correctly identifying all five toxinotypes (Figure 5). Furthermore, we evaluated the practicality of the Cp-MRC12a assay using clinical patient samples and spiked animal samples. The assay successfully detected all positive samples and accurately determined their toxinotypes (Figure 6), with results fully consistent with multiplex PCR (Supplementary Figure 2). The result of clinical sample detection is consistent with previous epidemiological data, confirming that *C. perfringens* type A is the most common toxinotype associated with human infections (Brynestad and Granum, 2002; Camargo et al., 2024; Gohari et al., 2021). These findings demonstrate that Cp-MRC12a is well-suited for reliable detection and typing of *C. perfringens* in real-world samples.

In conclusion, this study presents the first multiplex RPA-CRISPR/Cas12a assay for typing all five *C. perfringens* toxinotypes (A-E) with robust performance characteristics. The assay allows rapid detection, completing the process in approximately 65 min. It exhibits high sensitivity, with a detection limit of 10 copies/μL for type A and 100 copies/μL for types B-E, and shows good specificity, with no cross-reactivity with non-*C. perfringens* strains. The assay is simple to operate and does not require a thermal cycler. Results can be conveniently read by the naked eye using a portable fluorescence device, and can also be measured by a multifunctional microplate reader. The single-tube multiplex amplification reduces reagent consumption and improves cost-effectiveness (Supplementary Table 3), making the assay highly suitable for applications in resource-limited settings. Although the current work focused on a quadruplex RPA format, the Cp-MRC12a platform could be readily extended to include additional target genes, provided that the corresponding primer pairs efficiently amplify their targets. Unsurprisingly, while this manuscript is ready to submit the revised version, one piece of work was published on *Talanta*, which described the use of the RPA assay to amplify six target genes simultaneously in one tube and developed a rapid, equipment-free, and highly sensitive method to identify *C. perfringens* (Wang et al., 2026). Overall, Cp-MRC12a represents a promising tool for the rapid and accurate detection and typing of *C. perfringens* in humans and animals, with particular utility in resource-limited settings and field applications.

## Data Availability

The original contributions presented in this study are included in this article/Supplementary material, further inquiries can be directed to the corresponding authors.

## Footnotes

1
https://crispr.zhejianglab.com/index
