# Supplementary material for: A novel typing method for Clostridium perfringens using multiplex recombinase polymerase amplification and CRISPR/Cas12a
Source: Front Microbiol. 2026 Feb 18;17:1770883. doi: 10.3389/fmicb.2026.1770883 (PMC12957241; doi:10.3389/fmicb.2026.1770883)
Supplement: Supplementary file 1 [file Data_Sheet_1.docx]

Supplementary Materials for

**A Novel Typing Method for *Clostridium perfringens* Using Multiplex Recombinase Polymerase Amplification and CRISPR/Cas12a**

Siying Li,^1,2†^ Qinghong Zhou,^1†^ Qingxun Zhang,^3^ Ziqin Lin,^4^ Qianyi Zhang,^5^ Sihong Wu,^6^ Luying Wang,^1^ Sheng Ye,^1*^ Xingxing Xiao,^1*^ Shuai Gao^1*^

†These authors contributed equally to this work.

*Corresponding author. Sheng Ye, stevenyes@wmu.edu.cn; Xingxing Xiao, xiaoxingxing@wmu.edu.cn; Shuai Gao, 1359790915@qq.com.

**Supplementary Table 1**. The major lethal toxins produced by five types of *C. perfringens* and the most significant diseases associated with these bacteria.

| Toxin  type | Toxin produced | | | | Most significant diseases |
| --- | --- | --- | --- | --- | --- |
|  | α (*cpa*) | β (*cpb*) | ε (*etx*) | ι (*itx*) |  |
| A | + | – | – | – | Gas gangrene of humans and animals;  Food poisoning and non-foodborne gastrointestinal illness of humans;  Enterotoxemia of livestock;  Necrotic enteritis of poultry. |
| B | + | + | + | – | Lamb dysentery;  Necrotic enteritis and enterotoxemia of cattle and horse. |
| C | + | + | – | – | Human enteritis necroticans (Darmbrand, pigbel);  Hemorrhagic and necrotizing enteritis of several animals (cattle, sheep, horse, pigs). |
| D | + | – | + | – | Enterotoxemia in goats, sheep and cattle;  Enterocolitis in goats. |
| E | + | – | – | + | Possible association with gastroenteritis of sheep, cattle and rabbits. |

**Supplementary Table 2**. Sequences of primer pairs for multiplex PCR in this study

| Toxin | Gene | Primer sequence (5’-3’) | Product length (bp) |
| --- | --- | --- | --- |
| α | *cpa* | F: GCTAATGTTACTGCCGTTGA  R: CCTCTGATACATCGTGTAAG | 325 |
| β | *cpb* | F: GCGAATATGCTGAATCATCTA  R: GCAGGAACATTAGTATATCTTC | 196 |
| ε | *etx* | F: TGGGAACTTCGATACAAGCA  R: AACTGCACTATAATTTCCTTTTCC | 380 |
| ι | *itx* | F: AATGGTCCTTTAAATAATCC  R: TTAGCAAATGCACTCATATT | 272 |

**Supplementary Table 3. Cp-MRC12a cost analysis**

| **Component** | **Amount** | **Vendor (Cat #)** | **Cost ($)** | **Fraction**  **used/reaction** | **Cost/**  **reaction ($)** | **Cost/**  **Sample ($)** |
| --- | --- | --- | --- | --- | --- | --- |
| RPA | 1 kit | Qitian (B00000) | 114.29 | 5.21E-03 | 0.60 | 0.60 |
| Cas12a | 2000 pmol | NEB (M0653T) | 250 | 1E-03 | 0.25 | 1 |
| crRNA | 2400 pmol | Sangon Biotech | 51.43 | 8.33E-04 | 0.043 | 0.172 |
| Probe | 9420 pmol | Sangon Biotech | 17.71 | 5.31E-04 | 0.0094 | 0.0376 |
| Reaction buffer | 5 mL | NEB (B6002S) | 35.71 | 6E-04 | 0.021 | 0.084 |
| RNase-free Water | 500 mL | invitrogen (10977015) | 42.86 | 5.4E-05 | 0.0023 | 0.0092 |
|  |  |  |  | **Total ($)** | **0.9257** | **1.9028** |


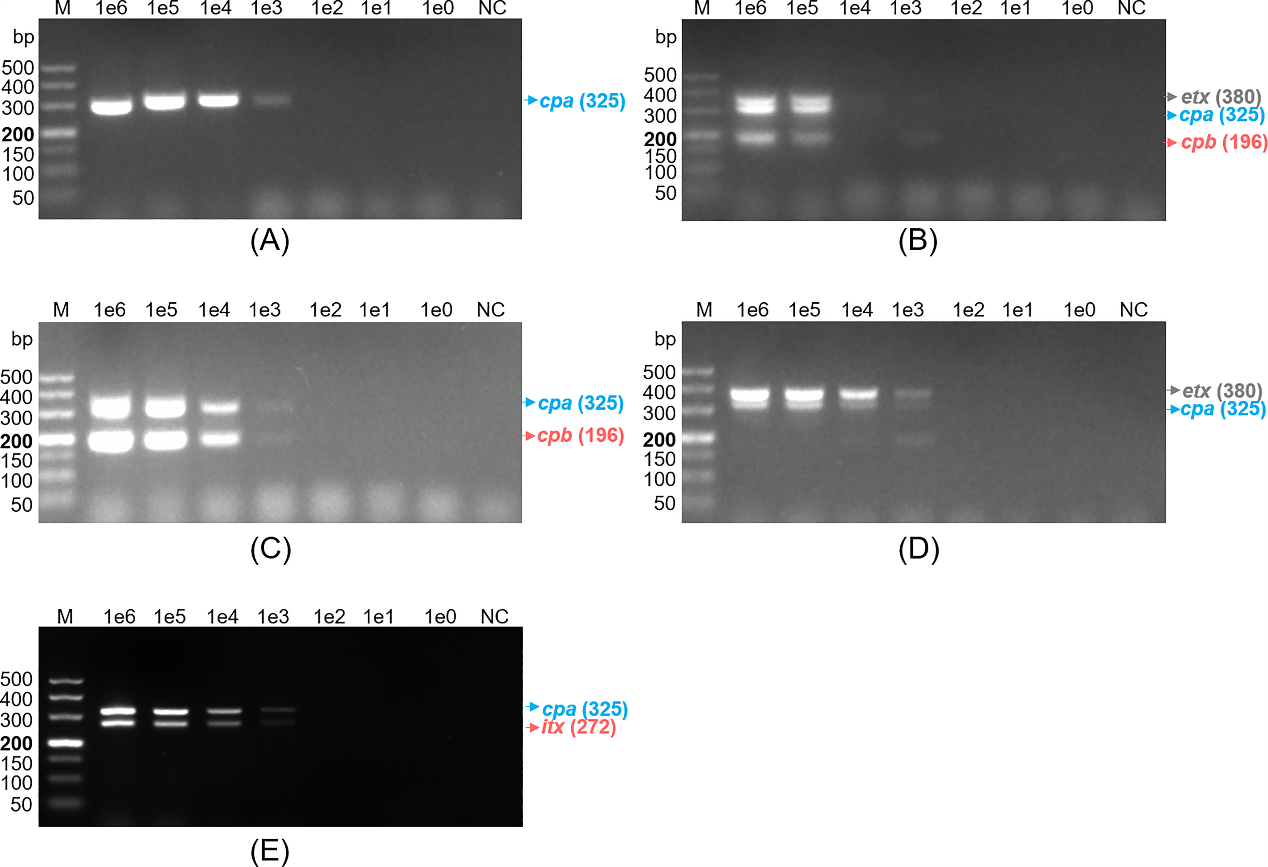


**Fig. S1**. Sensitivity evaluation of multiplex PCR assay in typing *C. perfringens*. Multiplex PCR assays were conducted using the different concentrations of genomic DNA of *C. perfringens* type A (A), type B (B), type C (C), type D (D), and type E (E) as templates, and H_2_O was used as the negative control (NC). The PCR products were analyzed using the 2% agarose gel electrophoresis. M, 500 DNA marker.


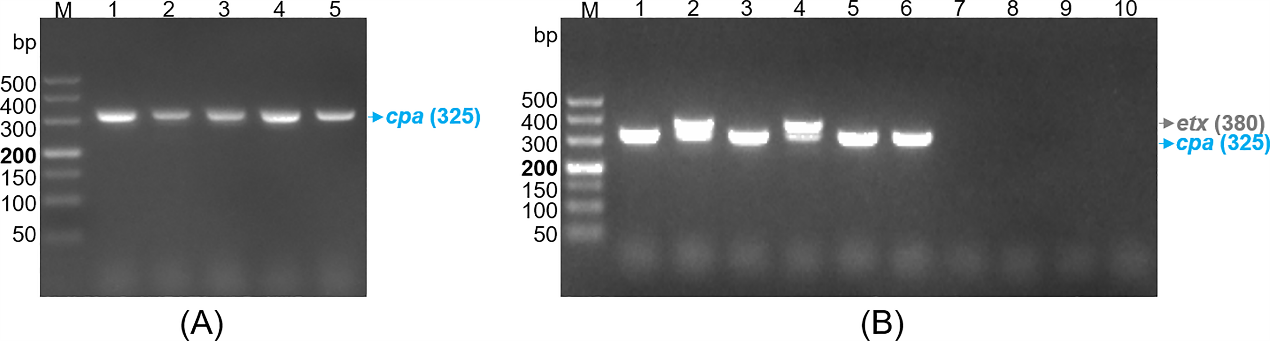


**Fig. S2**. Analyzing the clinical and spiked samples using the multiplex PCR assay. Five clinical samples (A) and six spiked samples (B) were analyzed by multiplex PCR. (A) 1-5 were five stool samples collected from five patients diagnosed with *C. perfringens* infection. (B) 1 and 6 were pig and chicken samples spiked with *C. perfringens* type A genomic DNA, 2 and 3 were cow samples spiked with genomic DNA of *C. perfringens* types A and D, respectively, 4 and 5 were sheep samples spiked with genomic DNA of *C. perfringens* types A and D, respectively, and 7-10 were four fecal samples collected from cattle, sheep, pig and chicken, respectively. The PCR products were analyzed using the 2% agarose gel electrophoresis. M, 500 DNA marker.
